# Contrast-Enhanced Ultrasound Improves the Pathological Outcomes of US-Guided Core Needle Biopsy That Targets the Viable Area of Anterior Mediastinal Masses

**DOI:** 10.1155/2018/9825709

**Published:** 2018-01-18

**Authors:** Jian-hua Zhou, Hong-bo Shan, Wei Ou, Yun-xian Mo, Jin Xiang, Yu Wang, Jian Li, Si-yu Wang

**Affiliations:** ^1^Department of Diagnostic & Interventional Ultrasound, Sun Yat-Sen University Cancer Center, State Key Laboratory of Oncology in South China, Collaborative Innovation Center for Cancer Medicine, Guangzhou, China; ^2^Department of Endoscopy, Sun Yat-Sen University Cancer Center, State Key Laboratory of Oncology in South China, Collaborative Innovation Center for Cancer Medicine, Guangzhou, China; ^3^Department of Thoracic Surgery, Sun Yat-Sen University Cancer Center, State Key Laboratory of Oncology in South China, Collaborative Innovation Center for Cancer Medicine, Guangzhou, China; ^4^Guangdong Association Study of Thoracic Oncology, Guangzhou, China; ^5^Department of Radiology, Sun Yat-Sen University Cancer Center, State Key Laboratory of Oncology in South China, Collaborative Innovation Center for Cancer Medicine, Guangzhou, China; ^6^Department of Pathology, Sun Yat-Sen University Cancer Center, State Key Laboratory of Oncology in South China, Collaborative Innovation Center for Cancer Medicine, Guangzhou, China; ^7^Department of Internal Medicine, Sun Yat-Sen University Cancer Center, State Key Laboratory of Oncology in South China, Collaborative Innovation Center for Cancer Medicine, Guangzhou, China

## Abstract

Based on the option that ultrasound-guided core needle biopsy (US-CNB) of the enhanced portion of anterior mediastinal masses (AMMs) identified by contrast-enhanced ultrasound (CEUS) would harvest viable tissue and benefit the histological diagnoses, a retrospective study was performed to elucidate the correlation between the prebiopsy CEUS and diagnostic yield of AMMs and found that CEUS potentially improved the diagnostic yield of AMMs compared with conventional US with a significant increase in the cellularity of samples. Furthermore, the marginal blood flow signals and absence of necrosis can predict the diagnostic yield of AMM. It was concluded that US-CNB of the viable part of AMMs, as verified by CEUS, was able to harvest sufficient tissue with more cellularity that could be used for ancillary studies and improve the diagnostic yield. And CEUS was recommended to those patients with AMMs undergoing repeated US-CNB, with the absence of marginal blood signals or presence of necrosis.

## 1. Introduction

Anterior mediastinal masses (AMMs) may appear in a wide variety of diseases from benign lesions to extremely malignant diseases. Masses in this area are more likely to be malignant than those in other compartments of the mediastinum. Lymphomas and thymic epithelial tumors are the two most common etiologies of AMMs [1]. Treatment strategies for AMMs are diverse and are based on a conclusive histological diagnosis with subclassification (such as medical treatment for lymphoma and neoadjuvant radiochemotherapy with surgery for advanced thymic epithelial tumors). Since it is the era of personalized medicine, strategies may also be based on genetic information (such as targeted therapy for non-small cell lung cancer based on testing for epidermal growth factor mutations and anaplastic lymphoma kinase rearrangement) [2].

Available approaches for the histological diagnosis of AMMs include the following: image-guided fine needle aspiration or core needle biopsy, endobronchial ultrasound-guided transbronchial or endoscopic ultrasound-guided transesophageal needle aspiration biopsy, and surgical procedures such as parasternal anterior mediastinotomy, cervical mediastinoscopy, video-assisted thoracoscopic surgery, and thoracotomy. In general, AMMs that are suspected to be malignant without upfront surgical resection are recommended for imaging-guided core needle biopsy [3, 4]. Related studies have demonstrated that satisfactory specimens can be obtained by core needle for a more accurate histological diagnosis with subclassification and genetic information for personalized therapy and prognosis [5–8].

The image guidance of computer tomography (CT) involves the use of radiation, is expensive, and lacks real-time monitoring, which means that it is an alternative approach for AMMs that cannot be adequately imaged by ultrasound (US) [9]. Evidence of the AMM by B-mode US is the first step for ultrasound-guided core needle biopsy (US-CNB). Vascular information can be obtained by color Doppler ultrasound, which helps to extend the diagnostic potential and safety of this minimally invasive procedure. The advantages of US guidance include real-time needle movement control, real-time blood flow imaging, minimal invasiveness, cost-effectiveness, and the ability to perform the biopsy procedure at the bedside when critically ill patients are in a semiupright position. Considering these advantages, US-CNB is the most efficient first-line approach for the biopsy of AMMs if the target is adequately imaged [10]. According to previous studies, the diagnostic yield of US-guided biopsy of AMMs varies from 70 to 90% [11–13]. The occasional failure of the diagnosis is primarily due to necrosis or fibrosis of the lesion, low cellularity, or sampling error [14]. Since it is difficult to identify these situations by conventional US, multiple punctures or repeated biopsies are performed to avoid a false-negative diagnosis and to increase the diagnostic yield, which increase the cost and delay therapy [15]. Fortunately, with the use of contrast agents, contrast-enhanced ultrasound (CEUS) offers an effective way to image tumor vascularity in both animal and clinical studies [16, 17]. CEUS patterns and features in the differentiation of malignant and benign diseases of the chest are controversial [18–21]. CEUS is not routinely performed for AMMs but is used on demand to address specific questions raised in an individual patient. In CEUS, the depiction of nonperfused areas (potentially necrotic, liquid, or fibrotic areas) might be relevant information prior to any US-guided biopsy [22]. This study is based on the hypothesis that the prebiopsy CEUS of AMMs will improve the delineation of viable from nonviable tissue and hence allow the targeting of the viable area of AMMs and the harvest of biopsy samples with more cellularity. This would ultimately lead to a conclusive histological diagnosis, which will benefit therapeutic decision-making.

This retrospective study aimed to compare the usefulness between conventional US and CEUS in their ability to identify the target area of AMMs and to plan the core needle biopsy route. Another study aim was to assess possible prebiopsy ultrasonic characteristics that may predict the patients with the highest probability of achieving conclusive histological diagnoses.

## 2. Patients and Methods

### 2.1. Study Population

The present study was approved by the Research and Ethics committee of Sun Yat-Sen University Cancer Center (SYSUCC), and written informed consent was obtained from each patient before CEUS and US-CNB were performed.

Masses located in the precardiac vascular region of the mediastinum were diagnosed as AMMs by the radiologist [23]. A total of 92 patients with AMMs suspected to be malignant that were detected by chest CT from July 2006 to June 2016 at our institution underwent initial US-CNB. The inclusion criteria for referral for US-CNB were based on the CT findings of a suspected AMM located adjacent to the chest wall and confirmed by conventional US evaluation. Since it is considered the shortest distance from the cutting system, solid content in the AMM should be at least 15 mm thick. Patients were able to control their breathing during the procedure. The International Normalized Ratio was not greater than 1.6 and the platelet count was greater than 10^5^/L.

The patients' demographic data, ultrasonic characteristics, diagnostic procedures, cost and duration between the initial US-CNB and treatment, hospitalization, pathological results, and clinical treatment records were reviewed using the Panoramic Patients Information System from the Department of Information.

### 2.2. Prebiopsy US and CEUS Evaluation

The conventional US evaluation of the AMMs included the B-mode of grey scale US and the C-mode of color Doppler blood flow. Grey scale US was used in the initial evaluation of the AMMs of patients who were recommended to undergo US-CNB. Location, size, ultrasonic pattern, and presence of necrosis were recorded. The color Doppler window was focused on the AMM to detect blood flow signals. The blood flow signals within the tumor were then categorized as “marked flow signals” or “not marked” including no, minimal, or moderate blood flow signals, or no-due to interference by the heart-beat, with reference to Adler's method [24]. The Doppler filter was adjusted on an individual basis to eliminate the influence from the heart-beat. Necrosis was determined if B-mode showed an echoic area with a clear boundary within the AMMs where the CDFI detected the absence of blood flow signals.

CEUS was performed with an Acuson Sequoia 512 (Siemens Medical Solutions, Mountain View, CA, USA) coupled to a 4C1 convex array probe using a low mechanical index (0.18) to avoid disruption of microbubbles. A 2.4 ml bolus of a US blood pool contrast agent (SonoVue, Bracco, Milan, Italy) was injected into the antecubital vein, followed by a 5-ml saline flush. Next, the AMM was scanned continuously for up to 4 minutes. The dynamic image was recorded on the hard-drive of the ultrasound system. Necrosis was determined if CEUS showed the complete absence of enhancement during all phases.

At the end of conventional US evaluation, or after supplement with CEUS, an appropriate approach to achieve a suitable acoustic window for the biopsy path and target was determined. The operator of the US-CNB should be involved in the evaluation of the CEUS procedure.

### 2.3. US-CNB

An ultrasonography system (Avius, Hitachi, Tokyo, Japan) with a 2.0–5.0 MHz ultrasound interventional probe (EUP-B512, Hitachi, Tokyo, Japan) was used for the biopsy, and color Doppler imaging was routinely used to delineate large vessels, such as the internal thoracic artery, that were in close proximity to the AMMs to avoid puncturing them during the biopsy. The information obtained from the diagnostic chest CT and prebiopsy US or CEUS was used to optimize and plan the biopsy route and target. An 18-gauge core biopsy needle (Magnum; Bard, Covington, GA, USA) was used for the transthoracic CNB. Children under 16 years of age were recommended to undergo this procedure in the operation theater with nonintubation general anesthesia; adults underwent this procedure after routine sterilization and local anesthesia (3–5 ml 1% lidocaine). A free hand approach was used for the CNB procedure. The probe was fixed and the core needle was inserted into the chest wall in the intercostal muscles. The core needle was fired until the tip of the needle reached the margin of the AMM. The whole procedure was monitored by real-time US. The number of puncture attempts was decided by the volume and quality of the specimen obtained. The specimens were fixed in 10% formalin and were sent to the pathology department for evaluation by 2 experienced pathologists. In some cases, half the specimen that was harvested was promptly collected in a sterile tube for molecular studies. The patients stayed in the recovery room for at least 30 minutes so that possible morbidities such as active bleeding or other complications could be observed.

### 2.4. Pathological Evaluation and Cellularity

Core needle biopsy specimens were stained with routine hematoxylin and eosin (H&E). All results that described staining patterns or morphologic features of the specimens were evaluated under the guidance of 2 experienced pathologists who specialized in cancer pathology. The ancillary study, which included immunohistochemistry (IHC), in situ hybridization (ISH), and fluorescence in situ hybridization (FISH) was performed for the requirements of the pathologist. Lung tissue found in the sample was noted. Diagnoses of lymphoma, thymoma, and carcinoma without accurate subclassification or origin were regarded as nonconclusive histological diagnoses. A histological diagnosis of normal, hyperplasia with fibrosis, necrotic tissue, low cellularity, or insufficient tissue was defined as a failed diagnosis. Failure and nonconclusive diagnoses were both regarded as nonconclusive histological diagnoses. A conclusive histological diagnosis was achieved by a pathologist with H&E staining and the required ancillary studies based on the CNB samples that were used in treatment decisions. The final diagnoses based on the definitive histological diagnoses obtained by the biopsy were confirmed by surgical pathology or response to medical treatment.

The cancer pathologist also selected the photomicrographs (magnification 40x) for the computer-assisted image analysis of cellularity (Axio Imager, Zeiss Imaging System, Germany). The percentage of tumor cells was evaluated in each biopsy sample. The primary endpoint was the maximum percentage of tumor cells across the different samples.

### 2.5. Statistics

SPSS software version 23.0 (IBM, Armonk, NY, USA) was used for all statistical analyses. The Mann–Whitney* U* test was used for numerical data, while the Pearson Chi-square test was used for categorical data. Binary logistic regression analysis was used to determine possible factors that could predict a conclusive histological diagnosis by US-CNB. Statistical significance was set at *p* < 0.05.

## 3. Results

### 3.1. General

**Figure 1:**
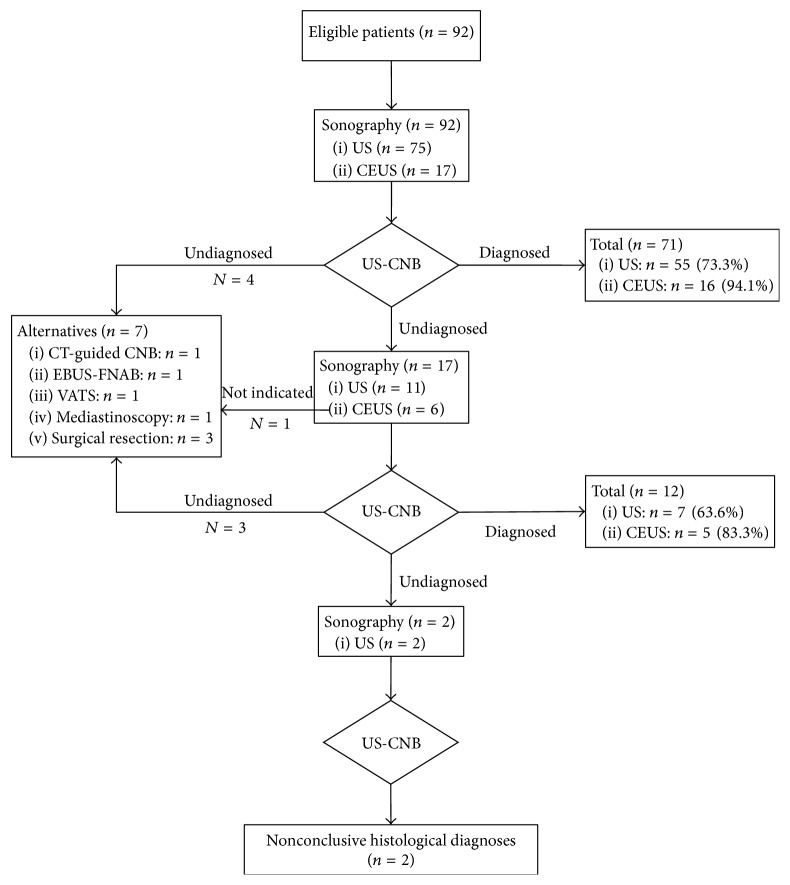
The flow chart of the 92 patients with anterior mediastinal masses who underwent initial ultrasound-guided core needle biopsy with prebiopsy ultrasound or contrast-enhanced ultrasound evaluation. CEUS: contrast-enhanced ultrasound; US: ultrasound; AMMs: anterior mediastinal masses; US-CNB: ultrasound-guided core needle biopsy.

Out of all patients, 64 were men and 28 were women, who had a mean age of 34.5 years (range: 5.0–68.0 years). All patients underwent evaluation by US (*n* = 75) or CEUS (*n* = 17) before the initial US-CNB. Eighteen of those 20 patients who were undiagnosed as a result of the initial US-CNB with prebiopsy US underwent US (*n* = 11) or CEUS (*n* = 7) evaluation before the repeated US-CNB. Two undiagnosed patients who underwent repeated US-CNB with prebiopsy US underwent multiple US-CNB procedures. In all, 308 punctures were performed (range: 2–5, mean: 2.8 punctures per patient). Seven patients who had undergone US-CNB and failed to receive a conclusive histological diagnosis were referred to other alternative procedures such as CT-guided biopsy, EBUS-TBNA, or a surgical procedure such as parasternal mini-mediastinotomy, cervical mediastinoscopy, VATS, or thoracotomy, as shown in Figure 1. The diagnostic yields of US-CNB according to the final diagnosis were as follows: 96.0% (24/25) for thymic epithelial tumors; 90.0% (9/10) for thymomas; 100.0% (15/15) for thymic carcinomas; 92.9% (39/42) for lymphomas; 60.0% (3/5) for Hodgkin's lymphomas; 97.3% (36/37) for non-Hodgkin's lymphomas that originated form T cells (*n* = 18) and B cells (*n* = 19); 90.0% (9/10) for germ cell tumors; 100% (2/2) for teratomas; 100.0% (1/1) for seminomas; 85.7% (6/7) for nonseminomatous or mixed germ cell tumors; 83.3% (10/12) for other malignancies; 66.7% (2/3) for metastases; 100% (3/3) for sarcomas; 75.0% (3/4) for lung cancers; 100.0% (2/2) for neuroendocrine tumors; and 100% (1/1) for tuberculosis.

### 3.2. Initial US-CNB for Histological Diagnoses of AMMs

No significant differences were observed in the demographic or ultrasonic characteristics including age, gender, cancer history, location, size, and CDFI category of AMMs between the US and CEUS groups. Prebiopsy CEUS detected more marginal blood flow signals and necrosis than conventional US (*p* < 0.05). Although no significant difference was observed in the number of punctures of the core needle between these two groups, the initial US-CNB with prebiopsy CEUS potentially improved the yield of conclusive histological diagnoses with increasing cellularity of the samples (*p* = 0.001); this helped to avoid repeated US-CNB compared with conventional US (*p* = 0.031), as shown in Table 1. The diagnostic yield of the initial US-CNB of AMMs was 77.2% (71/92).

### 3.3. Repeated US-CNB for the Histological Diagnoses of AMMs

**Figure 2:**
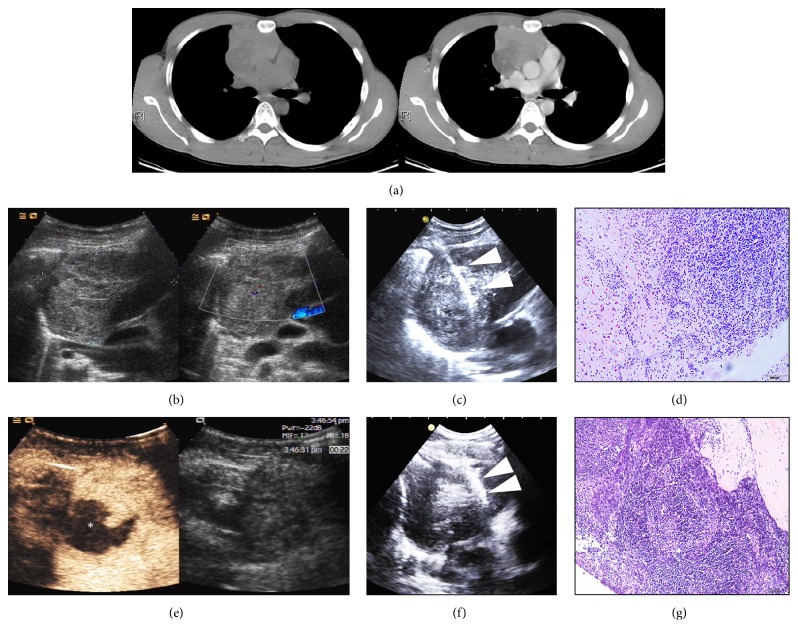
Twenty-nine-year-old man with thymoma. (a) Plain computed tomography revealed an irregular mass in the right anterior mediastinum. Contrast-enhanced computed tomography revealed that the mass was compressing the superior vena cava and aorta. (b) B-mode ultrasound showed an inhomogeneous mass visible in the right anterior mediastinum. Color Doppler ultrasound showed dot-like flow signals in the center of mass. (c) US-CNB of the mass with prebiopsy conventional US evaluation. White triangles indicate the needle. (d) H&E staining (magnification 100x) of the core needle biopsy sample showed major necrosis and a small number of enlarged nuclear cells with a nest-like arrangement, suspected tumor, and an insufficiency for immunohistochemistry staining. (e) Contrast-enhanced ultrasound revealed intensive inhomogeneous enhancement of the left anterior part of the AMM (22 seconds after the injection of 2.4 ml SonoVue); the left posterior part of the AMM was not enhanced throughout. The white flower-shaped dot indicates the necrosis with great confidence. (f) US-CNB of the mass with prebiopsy contrast-enhanced ultrasound targeted the left anterior enhanced portion of the AMM, which was confirmed by CEUS. (g) H&E staining (magnification 100x) of the core needle biopsy sample revealed karyomegaly within lymphocytes and a diagnosis of thymoma B1 with immunohistochemical staining, which was confirmed by surgical pathology. The approach of all ultrasonography procedures involved a right parasternal scan of the 3rd intercostal space.

Prebiopsy CEUS potentially improved the yield of conclusive histological diagnoses (5/6, 83.3%) compared with US (7/11, 63.6%) in individuals who underwent repeated US-CNB of AMMs (*p* = 0.395), as increased cellularity was observed in the samples (*p* = 0.001), as shown in Figure 2. Repeated US-CNB resulted in a diagnostic yield of 70.6% (12/17) and contributed to significant improvements in the diagnostic yield of those patients who underwent initial US-CNB with prebiopsy US from 73.3% (55/75) to 87.3% (67/75) (*p* < 0.001). Overall, the diagnostic yield of US-CNB increased from 77.2% (71/92) to 90.2% (83/92) with supplementation of repeated US-CNB in this study population (*p* < 0.001).

### 3.4. CEUS Improved the Diagnostic Yield of US-CNB

Taken together, prebiopsy CEUS improved the diagnostic yield (21/23, 91.3%) of US-CNB compared with prebiopsy US (62/86, 72.1%) (*p* = 0.043, odds ratio: 4.065, and 95% confidence interval: lower 0.885, upper 18.677) and decreased the need for repeated US-CNB in cases with a failed diagnosis (CEUS 0.0%, 0/2 versus US 75.0%, 18/24) and potentially avoided multiple US-CNB procedures (CEUS 0.0%, 0/1 versus US 40% 2/5).

### 3.5. Prebiopsy Ultrasonic Characteristics and Their Correlation with Histological Yield

A univariate analysis revealed that prebiopsy ultrasonic characteristics including marked blood flow signals (*p* = 0.004), the presence of marginal blood flow signals in close proximity to the probe (*p* = 0.002), and the absence of necrosis (*p* = 0.021) in the AMMs led to a higher conclusive histological diagnostic yield of US-CNB. Although more punctures (*p* < 0.001) and repeated procedures (*p* = 0.003) were performed, samples obtained from the patients with nonconclusive histological diagnoses were of low cellularity (*p* = 0.009), as shown in Table 2.

Logistic regression using the Enter method showed that prebiopsy ultrasonic characteristics including the presence of marginal blood flow signals (Exp(*B*) 0.116, 95.0% CI lower 0.021, upper 0.634) and the absence of necrosis (Exp(*B*) 5.986, 95.0% CI lower 1.185, upper 30.246) in AMMs can precisely predict the diagnostic yield (negative predictive value, 44.4%; positive predictive value, 97.6%; and overall predicative value, 92.4%).

### 3.6. Complications

Three patients complained of minor pain after completion of the procedure. No morbidities such as hemorrhage and pneumothorax were observed during or after the US-guided CNB procedure.

### 3.7. Treatment Based on the Conclusive Histological Diagnoses

The duration between the initial US-CNB and the treatment decision was shortened to a greater extent in those patients who underwent initial US-CNB with prebiopsy CEUS compared with those who underwent US (*p* < 0.001) because more repeated biopsies were needed in the conventional US group, which delayed the start of therapy. Of those 71 patients (71/92, 77.2%) who received conclusive histological diagnoses from the initial US-CNB, the results contributed to the best and prompt management decisions including those related to palliative care (*n* = 9), surgery-centered treatment (*n* = 6), chemotherapy-centered treatment (*n* = 55), and targeted therapy (*n* = 1) with crizotinib due to positive ALK gene translocation of lung cancer.

## 4. Discussion

AMMs were more likely to be malignant compared with masses in other parts of the mediastinum [1]. Successful biopsy of AMMs and the achievement of a conclusive histological diagnosis with subclassification or genetic information are crucial for prompt treatment decisions in the era of personalized therapy [25]. The present and previous studies showed high diagnostic yields and low morbidity in the group that underwent US-CNB for AMMs [14, 15, 19, 26, 27]. All of these studies approved US as a standard guidance for biopsy procedures if AMMs can be imaged well by US. We found that the therapeutic strategies based on the conclusive histological diagnoses after the initial US-CNB of AMMs were selected more promptly than in failed cases. Although repeated US-CNB increased the diagnostic yield, this procedure should be avoided due to high cost and time consumption, risk of complications, delayed therapy, and deterioration of the patients' faith in medicine and because repeated procedure is associated with increased anxiety and depression in patients [10].

Several studies have shown that CEUS can differentiate necrosis or nonviable tissue from viable tumor tissue with great confidence and that prebiopsy CEUS definitely improves the diagnostic yield of US-CNB of mediastinal masses. A prospective study with a small number of patients (15 patients) showed that B-mode US associated with CEUS and US-guided biopsy reached an elevated accuracy (91.66%, 11/12) for the diagnosis of mediastinal masses [19]. Most recently, several high-volume studies have demonstrated that, compared with conventional US, CEUS can improve the diagnostic accuracy of AMMs [26, 27]. The present study found that prebiopsy CEUS improved the diagnostic yield as a result of the pronounced ability of CEUS to distinguish viable tissue from necrotic or nonviable tissue. This was confirmed by the higher detection rate of nonenhancement area and higher cellularity in the CNB samples of those patients who underwent prebiopsy CEUS compared with those who underwent conventional US.

CEUS does not discriminate between benign and malignant tissue in pleural-based lesions or lung disease [18, 28]; the present study showed that CEUS contributes to the management of AMMs by US. CEUS plays a role in the exclusion of fully cystic lesions and in the selection of target areas in patients who are suitable for US-guided CNB. More patients (75.0%, 16/24) with failed diagnoses after US-CNB with prebiopsy conventional US underwent repeated US-guided CNB compared with those (0.0%, 0/2) with prebiopsy CEUS. Other biopsy alternatives but not repeated US-CNB should be recommended for those cases that failed to reach a conclusive diagnosis by US-CNB with prebiopsy CEUS, which means that CEUS even plays a role in the prevention of repeated US-CNB. No lung tissues were found in the CNB samples with prebiopsy CEUS without statistical significance; this may imply that prebiopsy CEUS could distinguish AMMs from surrounding atelectasis and avoid transpleural puncture, which is recommended for the diagnosis of thymic epithelial tumors according to the NCCN or ESMO guidelines [4, 29].

In the present study, the final diagnoses confirmed that the most common malignancies were lymphoma and thymic epithelial tumors. The diagnostic yield of US-CNB was higher in thymic epithelial tumors, but the diagnostic yield for Hodgkin's disease (60%, 3/5) was lower than that for non-Hodgkin's lymphoma (97.3%, 36/37). The present study also confirmed that an 18-gauge core needle biopsy for AMMs achieved a satisfactory yield for NHL (36/37, 97.3%) but not for HL (3/5, 60%). For the diagnosis of lymphoma, the recommendation is excisional biopsy, but core needle biopsy may be adequate if it is diagnostic [30, 31]. The diagnosis of Hodgkin's lymphoma depends on the presence of typical R-S cells and histological structure, which are always deficient in CNB samples. If Hodgkin's lymphoma is suspected, excisional biopsy is recommended [31]. Considering that non-Hodgkin's lymphoma is the main etiology of AMMs, core needle biopsy of the viable part of AMMs would contribute to the satisfactory pathological outcome and flow cytometry for the therapeutic strategy.

**Figure 3:**
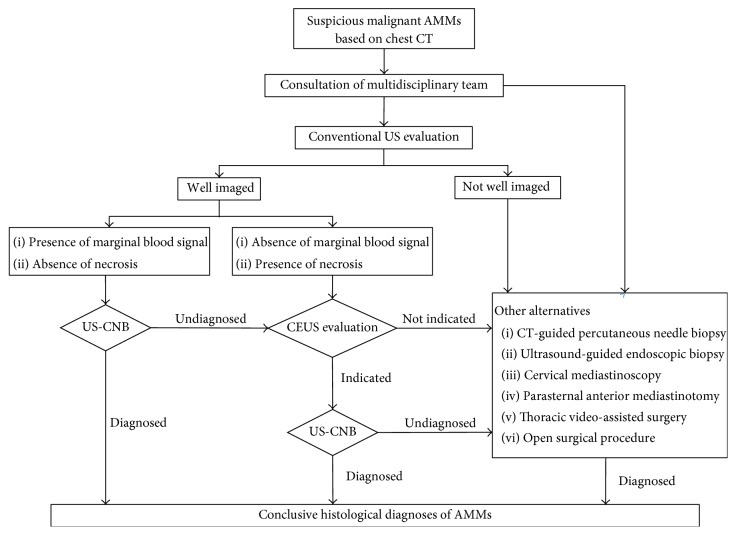
Suggested proposal for the integration of contrast-enhanced ultrasound into ultrasound management of suspicious malignant anterior mediastinal masses detected on chest computerized tomography. CEUS: contrast-enhanced ultrasound; US: ultrasound; AMMs: anterior mediastinal masses; US-CNB: ultrasound-guided core needle biopsy.

Conventional US evaluation and guided CNB can achieve a diagnostic yield as high as 70–90% [14, 26, 32]. Prebiopsy CEUS should not be routinely recommended for all patients with AMMs according to the cost-effectiveness principle. The present study found that prebiopsy ultrasonic characteristics of AMMs including the presence of marginal blood flow signals and the absence of necrosis can precisely predict the diagnostic yield. In addition, these 2 parameters could be used to triage the patients who underwent prebiopsy conventional US who may require further CEUS. Prebiopsy CEUS should be used selectively for AMMs with an absence of marginal blood flow signals or AMMs with necrosis or in those patients who undergo repeated CNB, just as the suggested proposal for integration of CEUS into the management of AMMs by US, as shown in Figure 3.

The present study is a retrospective review, and the low volume of prebiopsy CEUS procedures was not performed randomly, but on demand by the specialist. The study population included patients with AMMs that were suspected to be malignancies detected on chest CT; therefore, few patients with benign diseases were included. The dominant deficiency of the technique used in this study is that CEUS was not used directly for imaging guidance. It was used as part of a prebiopsy evaluation and supplied the operator with effective information to distinguish AMMs from the surrounding anatomical structures and to target the puncture area. Although the operators of US-guided CNB were involved in the CEUS evaluation, no precise spatial correlation was maintained between CEUS and CNB. The supposed proposal for integration of CEUS into the management of AMMs by US based on this study should be confirmed by random controlled trials that require multicenter cooperation for these scarce diseases of the anterior mediastinum.

## 5. Conclusion

US-CNB of the viable part of anterior mediastinal masses verified by prebiopsy CEUS supplies sufficient tissue with increased cellularity for underlying ancillary studies and increases pathologic yield. Further CEUS should be recommended for those AMMs with an absence of marginal blood flow signals close to the probe, those that are mostly necrotic, and patients who undergo repeated US-CNB.

## Figures and Tables

**Table 1:** Baseline characteristics, prebiopsy ultrasonographic features, and outcomes of 92 patients with AMMs who underwent initial US-CNB with prebiopsy conventional US or CEUS evaluation.

	US (*n* = 75)	CEUS (*n* = 17)	*p* value
Age (years), mean ± SD	34.0 ± 15.6	36.4 ± 17.1	0.763
Gender, male/female	50/25	14/3	0.207
Cancer history yes/no	3/75	2/17	0.205
Location of AMMs (both/left/right)	8/40/27	0/10/7	0.408
Size of AMMs (mm), mean ± SD	66.0 ± 29.3	70.1 ± 33.9	0.721
CDFI category (marked/not marked)	29/46	3/14	0.074
Marginal blood flow signals (presence/absence)	47/28	15/2	0.043
Necrosis (presence/absence)	8/67	5/12	0.046
Punctures of core needle (mean ± SD)	2.5 ± 0.8	2.9 ± 0.9	0.333
Repeated US-guided CNB (no/yes)	17/58	0/17	0.031
Conclusive histological diagnoses (no/yes)	20/55	1/16	0.067
Cellularity (mean ± SD)	0.64 ± 0.25	0.83 ± 0.18	0.001
Lung tissue in the sample (presence/absence)	6/69	0/17	0.144
Duration between initial CNB and treatment decision (days mean ± SD)	8.5 ± 4.2	5.9 ± 3.6	<0.001

US, ultrasound; US-CNB, ultrasound-guided core needle biopsy; AMM, anterior mediastinal mass; CEUS, contrast-enhanced ultrasound; SD, standard deviation.

**Table 2:** Results of the univariate analysis to establish confounding factors related to the ability to obtain a conclusive histological diagnosis of anterior mediastinal masses by US-CNB.

	Conclusive diagnoses (*n* = 83)	Nonconclusive diagnoses (*n* = 9)	*p* value
Age (years), mean ± SD	34.29 ± 16.36	36.00 ± 9.80	0.051
Gender, male/female	60/23	4/5	0.085
Cancer history (yes/no)	5/78	1/8	0.557
Location (both/left/right)	8/46/29	0/4/5	0.371
Size (mm), mean ± SD	81.6 ± 35.9	90.11 ± 29.78	0.819
CDFI category (marked/not marked)	58/25	2/7	0.004
Marginal blood flow signals (presence/absence)	60/23	2/7	0.002
Necrosis (presence/absence)	9/24	4/5	0.021
Punctures of core needle (mean ± SD)	3.3 ± 2.5	4.1 ± 2.0	<0.001
Repeated US-CNB (with/without)	12/71	5/4	0.003
Cellularity mean ± SD	0.7 ± 0.2	0.4 ± 0.3	0.009

US-CNB: ultrasound-guided core needle biopsy; CDFI: color Doppler flow imaging; SD: standard deviation.
